# Cyclic Equibiaxial Tensile Strain Alters Gene Expression of Chondrocytes via Histone Deacetylase 4 Shuttling

**DOI:** 10.1371/journal.pone.0154951

**Published:** 2016-05-05

**Authors:** Chongwei Chen, Xiaochun Wei, Zhi Lv, Xiaojuan Sun, Shaowei Wang, Yang Zhang, Qiang Jiao, Xiaohu Wang, Yongping Li, Lei Wei

**Affiliations:** 1 Department of Orthopaedics, the Second Hospital of Shanxi Medical University; Shanxi Key Lab of Bone and Soft Tissue Injury Repair, Taiyuan, Shanxi, China; 2 Department of Orthopaedics, Warren Alpert Medical School of Brown University/Rhode Island Hosptal, Providence, Rhode Island, United States of America; University of Maryland School of Medicine, UNITED STATES

## Abstract

**Objectives:**

This paper aims to investigate whether equibiaxial tensile strain alters chondrocyte gene expression via controlling subcellular localization of histone deacetylase 4 (HDAC4).

**Materials and Methods:**

Murine chondrocytes transfected with GFP-HDAC4 were subjected to 3 h cyclic equibiaxial tensile strain (CTS, 6% strain at 0.25 Hz) by a Flexcell^®^ FX-5000^™^ Tension System. Fluorescence microscope and western blot were used to observe subcellular location of HDAC4. The gene expression was analyzed by real-time RT-PCR. The concentration of Glycosaminoglycans in culture medium was quantified by bimethylmethylene blue dye; Collagen II protein was evaluated by western blot. Cells phenotype was identified by immunohistochemistry. Cell viability was evaluated by live-dead cell detect kit. Okadaic acid, an inhibitor of HDAC4 nuclear relocation, was used to further validate whether HDAC4 nuclear relocation plays a role in gene expression in response to tension stimulation.

**Results:**

87.5% of HDAC4 was located in the cytoplasm in chondrocytes under no loading condition, but it was relocated to the nucleus after CTS. RT-PCR analysis showed that levels of mRNA for aggrecan, collagen II, LK1 and SOX9 were all increased in chondrocytes subjected to CTS as compared to no loading control chondrocytes; in contrast, the levels of type X collagen, MMP-13, IHH and Runx2 gene expression were decreased in the chondrocytes subjected to CTS as compared to control chondrocytes. Meanwhile, CTS contributed to elevation of glycosaminoglycans and collagen II protein, but did not change collagen I production. When Okadaic acid blocked HDAC4 relocation from the cytoplasm to nucleus, the changes of the chondrocytes induced by CTS were abrogated. There was no chondrocyte dead detected in this study in response to CTS.

**Conclusions:**

CTS is able to induce HDAC4 relocation from cytoplasm to nucleus. Thus, CTS alters chondrocytes gene expression in association with the relocation of HDAC4 induced by CTS.

## Introduction

Chondrocytes are only cells in the articular cartilage to maintain the integrity of extracellular matrix, mainly including collagen and proteoglycans [1]. As the joint bearing loading, the chondrocytes are exposed to a combination of mechanical stress, in which tensile strain plays a critical role [2,3]. A large number of studies demonstrated that low-magnitude-and-frequency tensile strain has an anti-inflammatory function and promotes cartilaginous gene expression and matrix synthesis [3–6]; in contrast, high-magnitude-and-frequency tensile strain inhibites anabolism and induces catabolism as well as expression of inflammatory factor in chondrocytes [3,7,8]. However, the mechanisms by which the biomechanics, such as tension, regulates metabolism in cartilage are still not well understood.

Epigenetic evidence indicates that dynamic control of histone acetylation is an important physiological event of reversible post-translational modifiation in regulating gene expression [9]. Histone deacetylation by Histone deacetylase (HDACs) promotes chromatin condensation; however, histone acetylation by Histone acetylase (HATs) relaxes the structure of nucleosomes, which consequently alters interaction between histone and DNA, and controls gene transcription [10–12]. In mammalian cells, 18 HDACs have been discovered so far, which are divided into three major classes. Class I (HDAC1, 2, 3 and 8) and class III HDACs (consisting of a large family of sirtuins) are ubiquitously expressed; conversely, class II has a tissue-specific pattern of expression, which can be further divided into the following two subgroups: class IIa (HDAC4, 5, 7 and 9) and class IIb (HDAC6 and 10) [13]. Class IIa HDACs plays a significant role in tissue-specific growth and development[13–15]. HDAC4, a pivotal ingredient of the class IIa HDACs, is specifically expressed in the brain, heart, skeletal muscle and cartilage [13,16]. HDAC4-null mice display premature ossification of endochondrial bones due to ectopic and early onset chondrocyte hypertrophy, and then die early during the perinatal period [17]. HDAC4 has a unique ability of shuttling between the nucleus and cytoplasm [13,16]. Studies have shown that HDAC4 subcellular translocation plays a pivotal role in regulation of neuronal cell death [18], muscle cell differentiation [19], and growth plate chondrocyte differentiation [16]. However, whether biomechanics can regulate HDAC4 translocation between the cytoplasm and nucleus in chondrocytes remain unknown. We hypothesize that the biomechanics can regulate the gene expression of the chondrocytes via promoting HDAC4 relocation from cytoplasm to nucleus.

In this study we demonstrated that cyclic equibiaxial tensile strain (CTS, 6% strain at 0.25 Hz) could induce HDAC4 nuclear import, which resulted in increase of the gene expression of the anabolism and proliferation, and repression of hypertrophic gene expression.

## Materials and Methods

### DNA Constructs and Antibodies

Green fluorescent protein (GFP)-HDAC4 expression plasmids were provided by Miska EA [19]. The plasmids were cloned to recombinant adenovirus vectors, and the viral vectors were propagated in human embryonic kidney 293 cells. Viral infectious titers were 1×10^11^ pfu/mL. All antibodies were purchased from Santa Cruz Biotechnology (Santa Cruz, CA). The experimental protocol was approved by the Institutional Animal Care and Use Committee of the Second Hospital of Shanxi Medical University.

### Primary Cell Culture and Transfection

Chondrocytes were isolated from the ventral parts of the rib cages of 6-d-old newborn mice (C57Bl/6) and cultured in F-12 media with 10% fetal bovine serum (FBS) as previously described [20–22]. Briefly, the 6-d-old mice were killed with CO_2_, and the ventral parts of the rib cages were treated with 3% collagenase D (Roche, cat. no. 11 088 882 001). The chondrocytes were seeded at a density of 1×10^5^ cells/cm^2^ and cultured at 37°C in Ham’s F-12 medium with 10% FBS in a thermal incubator under 5% CO_2_. The medium of the chondrocyte culture was refreshed every other day.

Reaching 100% confluency, chondrocytes were subcultured into six-well BioFlex^®^ plates (25 mm diameter, Flexcell International Corporation, Hillsborough, NC) at a density of 45% confluency. After cultured for 12 hours, the culture medium was refreshed, and chondrocytes were incubated with adenoviral vectors containing GFP-HDAC4 for 24 hours at a multiplicity of infection (MOI) of 30. After 48 hours, transfection efficiency was confirmed with observation of the expression of GFP in infected chondrocytes by using Nikon E800 fluorescence microscope (Nikon, Melville, NY, USA).

### Application of Cyclic Equibiaxial Tensile Strain

**Fig 1:**
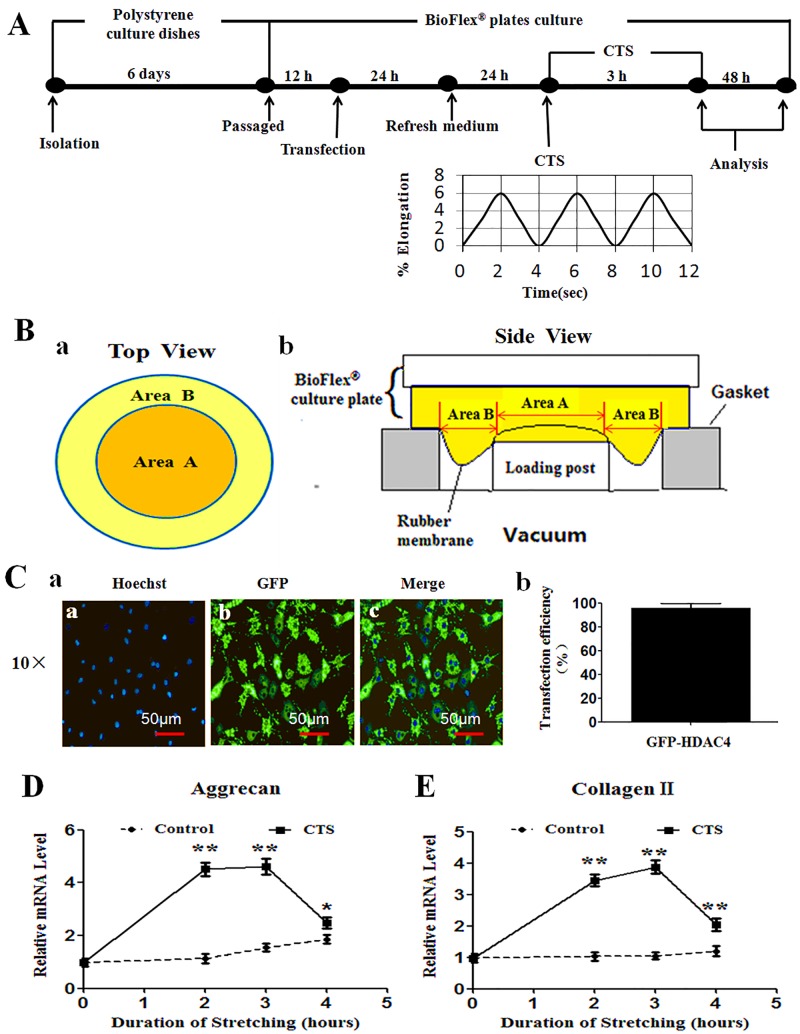
Overview of experimental design. (A) Workflow scheme for analysis of the effect of cyclic equibiaxial tensile strain (CTS) on HDAC4 relocation in chondrocytes. After isolated and then cultured for 6 days, the passage chondrocytes were transfected with GFP-HDAC4. At 48 h post-transfection, the transfected cells were submitted to CTS for 3 hours. (B) The chondrocytes at Area A were subjected to 6% equibiaxial CTS. (C) Green fluorescent protein (GFP) was captured by fluorescence microscope to validate the transfection efficiency of GFP-HDAC4 in chondrocytes. Nuclei were stained by Hoechst 33342 (blue) (C-a). 300 cells from 3 independent experiments were counted. Transfection efficiency of GFP-HDAC4 is 96%±3.64% (C-b). (D, E) The time-dependent changes in gene expression levels for aggrecan and type II collagen following CTS. Real-time PCR showed that mRNA level for aggrecan (D) and collagen II (E) were elevated after 2 and 3 h of CTS, but decreased after 4 h of CTS. Values are presented as mean±SD (n = 3). *P<0.05, **P<0.01 versus the non-stretched control group.

Chondrocytes were subjected to cyclic equibiaxial tensile strain (CTS), which consisted of sinusoidal equibiaxial strain from 0% to 6% amplitude at 0.25 Hz as indicated (Fig 1A), for 3 hours by using a computer-controlled Flexcell^®^ FX-5000^™^ Tension System (Flexcell International Corporation, Hillsborough, NC) as described in the manufacturer’s manual (www.flexcellint.com). To provide an equibiaxial strain, the six-well BioFlex^®^ culture plates were placed on a loading station. When vacuum was applied to a culture plate, the membrane deformation across the planar face of the loading post created uniform biaxial strain to the chondrocytes. As shown in the schematic diagram (Fig 1B), Area A above loading post was subjected to equibiaxial tensile strain (radial = circumferential strain), and Area B at the edge of the rubber membrane was provided for uniaxial strain (www.flexcellint.com). In this experiment, the cells at Area B were scraped off before tension. Thus the results from this experiment were the responses from the chondrocytes that were subjected to 6% equibiaxial CTS at Area A. The afore-mentioned method has been widely used [3,4,6,7,23]. Before mechanical application, the culture medium was replaced with 2 mL fresh serum-free Ham's F-12. In control group, chondrocytes were cultured equally in all steps, but no CTS was applied.

### Fluorescent Microscopy

To observe HDAC4 subcellular localization, chondrocytes were immediately incubated for 15 minutes with 10μg/mL of Hoechst 33342 (Pierce, Rockford, IL, USA) while protected from light after CTS. Stained nuclei (blue) and GFP-HDAC4 were examined with a Nikon E800 fluorescence microscope (Nikon, Melville, NY, USA).

### Evaluation of Cell Viability Following CTS

The viability of the chondrocytes subjected to CTS were evaluated by using Hoechst 33342 / Propidium Iodide (PI) Double Stain Apoptosis Detection Kit (Cat. L00309, GenScript, Piscatway, NJ, USA). Fourty-eight hours after CTS, the chondrocytes were incubated with Hoechst 33342 for 10 minutes at room temperature with avoidance of exposure to light; then the dye reagent (containing 1000 μL of 1× buffer A and 5 μL of PI prepared according to the manufacturer’s instruction) was added to each sample. After 10 min incubation, fluorescence microscope was used to capture the images of nuclear fluorescence stain (Nikon, Melville, NY, USA). Cell viability was quantified by scoring the dead (red) cells in proportion to the total ones. Chondrocytes that had been frozen at -20°C and thawed were served as positive controls.

### Nuclear and Cytosol Proteins Extracted and Western Blot Analysis

Chondrocytes were lysed with RIPA buffer (50 mM Tris·HCl, pH 8.0, 150 mM NaCl, 5 mM EDTA, 1% NP-40) at 4°C for 30 min. The nuclear and cytosol proteins from the chondrocytes were separated with the Nuclear Extract Kit (catalog no. 40010, Active Motif, Carlsbad, CA) following the manufacturer’s instructions. The BAC Protein Assay Reagent Kit (Pierce, Rockford, IL, USA) was used to quantify the total protein.

Western blot was carried out according to standard procedures. The protein was electrophoresed in 10% PAGE and transferred onto the Immobilon-Polyvinylidene Difluoride (PVDF) membrane. Anti-HDAC4 antibody (sc-46672, Santa Cruz), anti-histone 3 (sc-8655, Santa Cruz) or GAPDH (sc-47724, Santa Cruz) were used as primary antibodies at a concentration of 0.2μg/mL. Peroxidase-conjugated goat anti-mouse (sc-2005, Santa Cruz) or mouse anti-goat (sc-2354, Santa Cruz) were used as the secondary antibody, at 1:1000 dilution. Immunoreactive protein was visualized by using the ECL western blot detection reagents, and the membrane was exposed to Molecular Imager (Bio-Rad, Hercules, CA, USA). Band densities were quantified by using Image Acquisition and Analysis Software (UVP, Upland, CA, USA).

### Real-time RT-PCR

Total RNA was extracted from chondrocytes by using RNeasy Mini kit (Qiagen) and reversely transcribed into cDNA by using PrimeScript RT Master Mix kit (Takara, Tokyo, Japan) following the manufacturer’s instructions. Real-time PCR was performed by using SYBR Premix Ex TaqTM (Takara, Tokyo, Japan) according to the manufacturer’s instructions. The reaction conditions included denaturation at 95°C for 15 sec, 35 cycles at 95°C for 10 sec and 58°C for 30 sec. Non-specific amplification had not been determined by the dissociation curve. 18S mRNA served as an internal reference to normalize the levels of gene expression for all samples. The primer sequences were as follows. The forward and reverse primers for Aggrecan were 5'-CAG TGG GAT GCA GGC TGG CT-3' and 5'-CCT CCG GCA CTC GTT GGC TG-3', respectively; Collagen II were 5'-AAG GGA CAC CGA GGT TTC ACT GG-3' and 5'-GGG CCT GTT TCT CCT GAG CGT-3', respectively; Polo-like kinase 1 (LK1) were 5'-CCG CCT CCC TCA TCC AGA AG-3' and 5'- GCG GGG ATG TAG CCA GAA GTG-3', respectively; SOX-9 were 5'- CGT GGA CAT CGG TGA ACT GA -3' and 5'-GGT GGC AAG TAT TGG TCA AAC TC-3', respectively; Runx2 were 5'-CCG CAC GCA AAC CGC ACC AT-3' and 5'-CGC TCC GGC CCA CAA ATC TC-3', respectively; Ihh were 5'-CCA CTT CCG GGC CAC ATT TG -3' and 5'-GGC CAC CAC ATC CTC CAC CA-3', respectively; MMP-13 were 5'-GGA CCT TCT GGT CTT CTG GC-3' and 5'-GGA TGC TTA GGG TTG GGG TC-3', respectively; Collagen X were 5'-GCC AGG AAA GCT GCC CCA CG-3' and 5'-GAG GTC CGG TTG GGC CTG GT-3', respectively; 18S rRNA were 5'-CGG CTA CCA CAT CCA AGG AA-3' and 5'-GCT GGA ATT ACC GCG GCT-3', respectively.

The cycle threshold value for target gene was measured and calculated by computer software IQ50 (Bio-Rad Laboratories, Hercules, CA, USA). Relative mRNA level was calculated as *x* = 2^-ΔΔCt^, in which ΔΔCt = ΔCt E - ΔCt C, ΔCt C = CtC-Ct18S, and ΔCt E = Ctexp-Ct18S [24].

### Glycosaminoglycan concentration in culture medium

Culture media were collected at 48 h post-CTS. Bimethylmethylene blue dye (DMMB) (Sigma) was used to spectrophotometrically quantify the sulfated glycosaminoglycan. Bovine chondroitin sulfate (Sigma) served as standard controls [25].

### Immunohistochemistry

To confirm that the chondrocytes subjected to CTS were not dedifferentiated, immunohistochemistry was performed with type II collagen and type I collagen staining. Cells were fixed with 4% paraformaldehyde for 15 min at 48 h post-CTS. The cells were incubated with primary antibodies against type II collagen and type I collagen for 1 h at 37°C (SC-25974, SC-7764, Santa Cruz, CA, USA). Then the cells were incubated with a Texas Red-conjugated secondary antibody (SC-2783, Santa Cruz, CA, USA) for 30 min. Cellular nucleus were stained with Hoechst 33242 (Pierce, Rockford, IL, USA). The image was captured by using a Nikon E800 fluorescence microscope.

### Okadaic Acid Inhibition Experiment

Okadaic acid (OA) (Sigma), a inhibitor which blocks the nuclear import of HDAC4 [26,27], was prepared as a 10-μM stock in dimethyl sulfoxide (DMSO, Sigma) and added to culture medium 2 hours before CTS at the final concentration of 10 nM [27,28].

### Statistical analysis

Each experimental measure was performed in triplicate. The data were obtained from at least three independent experiments, and expressed as means±SD. Two-way ANOVA was used to compare the time-dependent changes in mRNA levels of aggrecan and type II collagen. Two-tailed paired t-test was used to compare the data from two different groups. The data from three different groups were analyzed by one-way analysis of variance with multiple pair-wise comparisons made by the Student-NewmanKeuls method (three comparisons). P<0.05 was considered significant.

## Results

### Transfection Efficiency and Pilot Study

#### Transfection efficiency of GFP-HDAC4

**Fig 2:**
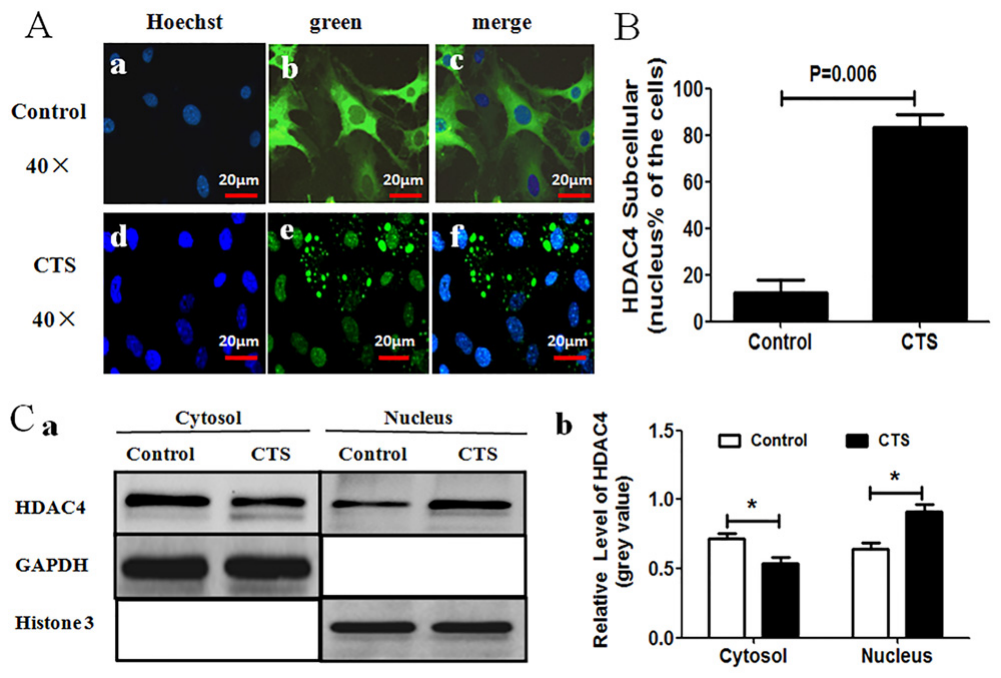
CTS-induced HDAC4 nuclear relocation in chondrocytes. (A) Fluorescence microscope showed that GFP-HDAC4 was mainly located in the cytoplasm of cells in non-stretched control group (a-c), while GFP-HDAC4 was relocated to nucleus of cells subjected to CTS (d-f). Green indicated the GFP-HDAC4 and blue indicated cell nuclei stained by Hoechst 33342. (B) Percentage of GFP-HDAC4 located in nucleus was scored. 300 cells from 3 independent experiments were counted. Data were expressed as means±SD (P = 0.006). (C) Nuclear and cytoplasmic lysates were separated and followed by western blot analysis with anti-HDAC4 antibody. Histone 3 and GAPDH acted as loading controls for the nuclear and cytoplasmic fraction respectively (C-a). Semi-quantitative assay of band densities showed that cytoplasmic HDAC4 was decreased, and nuclear HDAC4 was increased in CTS group as compared to non-stretched control group (C-b), Values were presented as mean±SD (n = 3).

To detect the efficiency of adenovirus-mediated transfection of GFP-HDAC4, chondrocytes were viewed under a fluorescent microscopy after 48 h of transfection. The efficiency of adenovirus mediated GFP-HDAC4 transfection was 96%±3.64% (Fig 2C-a and 2C-b).

#### A pilot study

To determine the duration of the CTS protocol which was necessary to promote highest gene expression in chondrocytes, a pilot study was performed to observe the time-dependent changes in gene expression of aggrecan and collagen II following CTS. Real-time PCR results demonstrated that mRNA level of two genes was significantly upregulated after 2 and 3 h of CTS, but decreased after 4 h of CTS (P<0.05 versus unloaded group) (Fig 1D and 1E). Therefore, all subsequent experiments were performed with a protocol of stretching 3 h duration.

### CTS Induced HDAC4 Nuclear Import

To investigate the effect of CTS on HDAC4 subcellular localization, the intracellular localization of GFP-HDAC4 was observed by fluorescence microscope after 3 h CTS protocol. Interestingly, we found that HDAC4 was mainly located in the cytoplasm of chondrocytes in non-stretched control group (Fig 2A-a to 2A-c), while most of the HDAC4 was relocated in the nuclei of cells which had undergone the CTS (Fig 2A-d to 2A-f). The percentage of chondrocytes that had HDAC4 located in their nuclei was higher in the CTS group than in the non-stretched control group (P = 0.006) (Fig 2B). To further prove the relocation of HDAC4 from the cytoplasm to nucleus occurred in response to CTS, Nuclear and cytoplasmic lysates were separated by using the Nuclear Extract Kit, and the protein levels of HDAC4 were detected by western blot analysis. The HDAC4 level in the cytoplasmic fraction was far higher in the non-stretched control group vs. CTS group; In contrast, the HDAC4 level in the nuclear fraction was less in control group than in the CTS group (Fig 2C-a and 2C-b). These results demonstrated that CTS induces HDAC4 relocation from cytoplasm to nucleus.

### CTS Regulates Gene Expression of Chondrocytes

**Fig 3:**
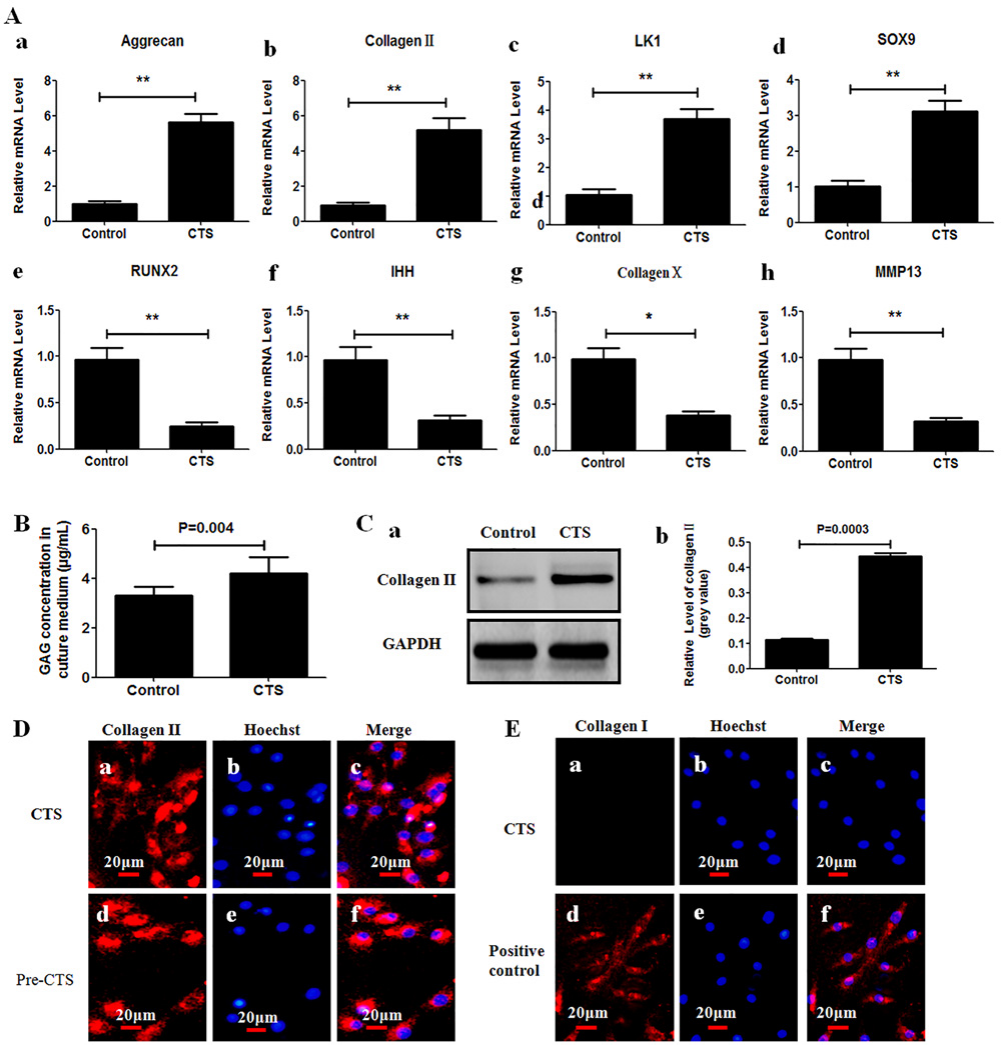
CTS induces anabolism and inhibits differentiation in chondrocytes. (A) The mRNA levels for aggrecan (a), collagen II (b), LK1 (c), SOX9 (d), Runx2 (e), Ihh (f), collagen X (g) and MMP-13 (h) were compared between chondrocytes subjected to CTS and non-stretched control cells. Values were presented as mean±SD (n = 3). *P<0.05, **P<0.01 versus the non-stretched control group. (B) CTS increased production of glycosaminoglycans. Glycosaminoglycans concentration in culture medium was higher in CTS group than that in control group at 48 h post-CTS. (C) CTS increased protein levels for collagen II. Western blot analysis showed a strong band in CTS group (C-a). Semi-quantitative assay of band densities showed that relative grey value of collagen II protein were higher in the CTS group than that in the control group at 48 h post-CTS(C-b). (D, E) Immunohistochemistry was performed to detect cells phenotype. (D) The cells were both found positive for collagen II at 48 h post-CTS and prior-to-CTS. (E) no chondrocyte was found positive for collagen I at 48 h post-CTS; Skin fibroblasts obtained from newborn mice (C57Bl/6) served as positive control.

To determine the effect of CTS on gene expression of chondrocytes, real-time PCR was performed to quantify mRNA levels in chondrocytes. It has been found that levels of mRNA for aggrecan, collagen II, LK1 and SOX9 were increased in cells subjected to CTS as compared to the non-stretched control cells (Fig 3A-a to 3A-d), which suggested that CTS had a positive effect on anabolism and proliferation of chondrocytes. On the contrary, the levels of mRNA for Runx2, Ihh, collagen X, MMP-13 were lower in cells subjected to CTS, as compared to control cells (Fig 3A-e to 3A-h), which indicated that CTS also inhibited differentiation of chondrocytes.

To further identify the effect of CTS on production of matrix protein, a portion of cells were cultured for additional 48 h after CTS at 37°C and 5% CO_2_, and subjected to further analysis. Increase of glycosaminoglycans concentration was detected in culture medium of CTS group as compared to the non-stretched control group (P = 0.004) (Fig 3B). Western blot analysis showed that relative grey value of collagen II protein was higher in CTS group than that in control group (P = 0.0003) (Fig 3C-a and 3C-b). These findings suggest that CTS was also able to enhance production of collagen II and glycosaminoglycans protein. Immunohistochemistry analysis showed a positive stain for collagen II (red) (Fig 3D), but not for collagen I (Fig 3E-a to 3E-c). These verified that the CTS protocol used in this study did not result in dedifferentiation of chondrocytes.

### OA Impairs the HDAC4 Nuclear Import

**Fig 4:**
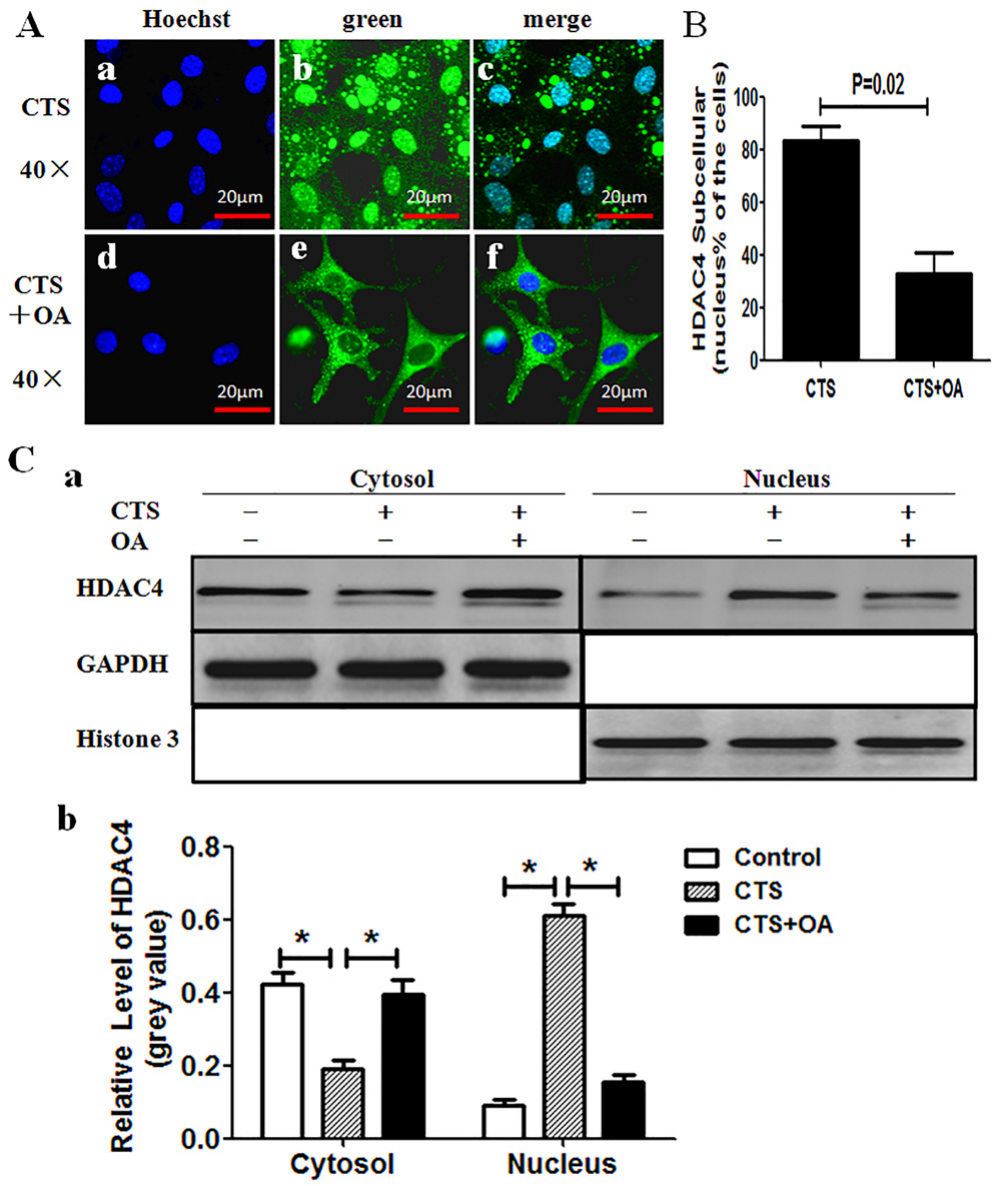
OA impairs the HDAC4 nuclear import induced by CTS. (A) Fluorescence microscope showed GFP-HDAC4 was mainly located in the nucleus in cells subjected to CTS only (a-c), and mainly in the cytoplasm in cells subjected to CTS with OA (d-f). Green indicated the GFP-HDAC4, and blue indicated cell nucleus stained by Hoechst 33342. (B) Percentage of green GFP-HDAC4 located only in nucleus was scored. 300 cells from 3 independent experiments were counted. Data were expressed as means±SD (P = 0.02). (C) Nuclear and cytoplasmic lysates were separated and followed by western blot analysis with anti-HDAC4 antibody. Histone 3 and GAPDH acted as loading controls for the nuclear and cytoplasmic fraction respectively (C-a). Semi-quantitative assay of band densities showed that cytoplasmic HDAC4 was decreased, and nuclear HDAC4 was increased in CTS group as compared to control group and CTS with OA group; The relative grey value of HDAC4 had no statistics difference between control group and CTS with OA group in both cytoplasm and nucleus (C-b). Values were presented as mean±SD (n = 3), *P<0.05.

**Fig 5:**
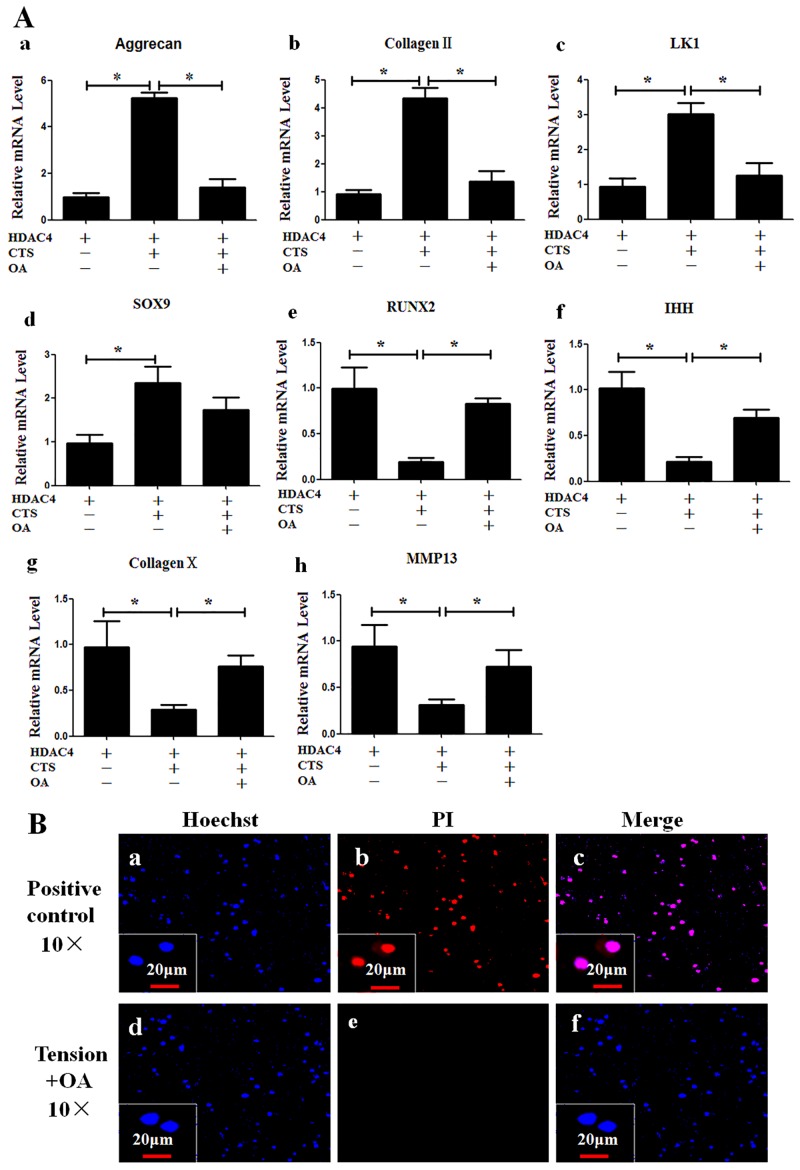
Inhibition of HDAC4-nuclear-import by OA abrogates the CTS-induced change of gene expression. (A) Real-time PCR was carried out to analyze the mRNA levels for aggrecan (a), collagen II (b), LK1 (c), SOX9 (d), Runx2 (e), Ihh (f), collagen X (g) and MMP-13 (h) in non-stretched without OA control group, CTS group and CTS with OA group. Values were presented as mean±SD (n = 3). *P<0.05. (B) OA did not induce cell death. The viability of cells subjected to CTS with OA was assessed by using Hoechst 33342/PI double staining at 48 h post-CTS. The cells frozen at -20°C served as positive controls (B-a to c). No dead cells were detected in CTS with OA groups (B-d to f). Blue indicated cell nucleus stained by Hoechst 33342, while red indicated PI stain in dead cells.

To further detect the relationship between HDAC4 nuclear relocation and gene expression of chondrocytes, as a first step, we observed whether OA inhibits HDAC4 nuclear import by fluorescent microscope. The chondrocytes were simultaneously subjected to CTS with or without OA. Just as expected, OA abrogates the CTS-inducing nuclear relocation of HDAC4 (Fig 4A and 4B). HDAC4 was mainly located in the nuclei of chondrocytes subjected to CTS without OA (Fig 4A-a to 4A-c); on the contrary, HDAC4 was mainly located in the cytoplasm of chondrocytes subjected to CTS with OA (Fig 5A-d to 5A-f). The percentage of chondrocytes that had HDAC4 located in their nuclei was higher in the groups subjected to CTS than in the groups subjected to CTS with OA (P = 0.02) (Fig 4B).

Subsequently, Nuclear and cytoplasmic lysates were separated, and western blot was performed to investigate the effect of OA on the location of HDAC4 between cellular cytosol and nucleus in three groups: first group subjected to non-stretched without OA drug (control group), second group to CTS, and third group to CTS with OA. Cytosol HDAC4 levels were far lower in CTS group when compared with the other two groups. In contrast, nuclear levels of HDAC4 were higher in CTS group than the other two groups. There was no statistical difference in cytoplasmic or nuclear fractions between control group and CTS with OA group.(Fig 4C-a and 4C-b).

### OA Abrogates the Effect of CTS on mRNA Expression

**Fig 6:**
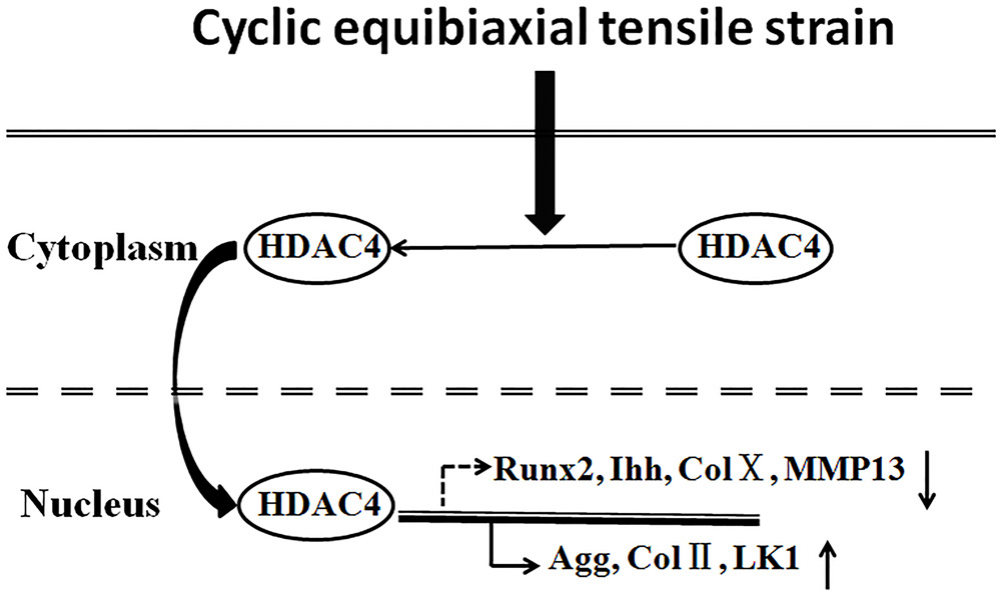
A model of HDAC4 cytoplasmic-nuclear relocation involved in gene expression in response to CTS. CTS induced HDAC4 relocation from cytoplasm to the nucleus. When HDAC4 relocated to the nucleus, it increases chondrogenic gene expression and reduces hypertrophic gene expression in the chondrocytes.

To verify whether the blocking of HDAC4-nuclear-import by OA also abolished the increase of gene expression that occured in response to CTS, mRNA levels of chondrocytes from control (non-stretched without OA), CTS, and CTS with OA groups were quantified using real-time PCR. The mRNA expression of aggrecan, collagen II and LK1 were all decreased in the cells subjected to CTS with OA when compared with CTS alone (Fig 5A-a to 5A-c); likewise, the levels of mRNA for Runx2, Ihh, collagen X and MMP-13 were all increased in the group subjected to CTS with OA compared with CTS only (Fig 6A-e to 6A-h).

We further confirmed that CTS with OA (10 nM) did not lead to chondrocyte death as detected by using Hoechst 33342 / PI double stain (Fig 6B).

## Discussion

When cartilage is exposed to mechanical stress, chondrocytes will be stretched by collagen and other extracellular matrixes that are linked to the chondrocytes. During physiological movements in vivo, articular chondrocytes were subjected to 5% tensile strain [5,8]. Evidences show that CTS with a magnitude of 6% is anti-inflammatory and promotes anabolism [6,29]; furthermore, 6% CTS do not lead to deformation of chondrocytes over a period of 6 days [5]. Studies demonstrate that exposure of chondrocytes to 0.25 Hz CTS is anti-inflammatory and promotes aggrecan synthesis [6]. Thus, the use of 6% CTS at 0.25 Hz in our experiments is close to the magnitude and frequency that chondrocytes experienced under physiologic conditions.

It is well known that mechanical stress is able to modulate chondrocyte functions [2]. Nevertheless, the mechanisms by which mechanical stimulation regulates chondrocyte functions remain largely unknown. Extensive studies demonstrate that HDAC4 subcellular translocation plays a key role in cell differentiation[14,19,30]. Studies [16] have also shown that HDAC4 regulates growth plate chondrocyte differentiation through relocation from the nucleus to the cytoplasm. However, it is still unknown whether HDAC4 subcellular translocation is regulated by mechanotransduction. In this study, for the first time, we found that HDAC4 could relocate from the cytoplasm to the nucleus in response to cyclic equibiaxial tensile strain (6%, 0.25 Hz) in chondrocytes. We further discovered that HDAC4 nuclear relocation suppressed the gene expression of Runx2, Ihh, collagen X and MMP-13, and increases the expression of aggrecan, collagen II, LK1 and SOX9. However, when OA drug impaired the HDAC4 nuclear import induced by CTS, the effect of CTS on mRNA expression was abrogated as well. This suggested that CTS altered gene expression is associated with the relocation of HDAC4 induced by CTS.

Previous studies have shown that HDAC4 controls chondrocyte hypertrophy by binding to and inhibiting Runx2 [17,31]. It is well known that Runx2 regulates chondrocyte differentiation by inducing the expression of Ihh, collagen X and MMP-13 [32–35]. Our previous studies [16] demonstrated that CaMKIV enhanced Runx2 promoter activities to modulate chondrocyte differentiation through HDAC4 subcellular translocation from the nucleus to cytoplasm during growth plate development. Our recent studies [36,37] showed that HDAC4 inhibited Runx2 promoter activities in a dose-dependent manner. Overexpression of HDAC4 decreased the expression of Runx2, Ihh, collagen X, MMP1, MMP3, MMP-13, ADAMTS-4 and -5, and increased the expression of aggrecan and collagen II [37]. In this study, we demonstrated that the HDAC4 nuclear relocation could inhibit the expression of Runx2, Ihh, collagen X and MMP-13. The mechanism behind it may be that relocating into nucleus from cytoplasm, HDAC4 would bind to Runx2 and inhibit chondrocyte differentiation. Thus, when the nuclear relocation of HDAC4 was abolished by OA drug, the decrease of Runx2, Ihh, collagen X and MMP-13 mRNA expression was also abrogated. This may partially explain why overexpression of HDAC4 inhibited expression of Runx2 and other downstream genes.

Interestingly, in addition to inhibiting chondrocyte differentiation, we noticed that subcellular relocation of HDAC4 also increased expression of aggrecan and collagen II. Previous studies showed that mechanical loadiong increases cartilaginous gene expression, such as aggrecan and collagen II [2]. However, whether the HDAC4 nuclear relocation is involved in the increase of cartilage matrix is unknown. Here we revealed that CTS improves gene expression of aggrecan and collagen II via promoting HDAC4 nuclear import.

In conclusion, our study presents a mechanism for CTS regulation of gene expression of chondrocytes, which is associated with HDAC4 relocation from the cytoplasm to nucleus (Fig 6). When HDAC4 relocates to the nucleus induced by CTS, it increases chondrogenic gene expression and reduces hypertrophic gene expression in the chondrocytes.
